# *Fusarium* Species Causing Pepper Wilt in Russia: Molecular Identification and Pathogenicity

**DOI:** 10.3390/microorganisms12020343

**Published:** 2024-02-06

**Authors:** Irina Engalycheva, Elena Kozar, Svetlana Frolova, Svetlana Vetrova, Tatyana Tikhonova, Elena Dzhos, Myazar Engalychev, Vera Chizhik, Viktor Martynov, Andrey Shingaliev, Ksenia Dudnikova, Maksim Dudnikov, Yulia Kostanchuk

**Affiliations:** 1 Federal State Budgetary Scientific Institution Federal Scientific Vegetable Center (FSBSI FSVC), 143072 Vniissok, Russia; kozar_eg@mail.ru (E.K.); svetlanaleonidovna95@gmail.com (S.F.); lana-k2201@mail.ru (S.V.); tikhonova@tatyana94.ru (T.T.); elenadzhos@mail.ru (E.D.); myazar@mail.ru (M.E.); chizhikvera@bk.ru (V.C.); 2 State Budgetary Scientific Institution Federal, All-Russian Research Institute of Agricultural Biotechnology (FSBSI ARRIAB), 127550 Moscow, Russia; martynov.vik@gmail.com (V.M.); kronstein491@yandex.ru (A.S.); saenkok1997@yandex.ru (K.D.); max.dudnikov.07@gmail.com (M.D.); 3 Federal Research Center of Biological Plant Protection, 350039 Krasnodar, Russia; 4 Federal State Budget Scientific Institution, Research Institute of Agriculture of Crimea, 295034 Simferopol, Russia; kostanyulya@mail.ru

**Keywords:** *Fusarium*, pepper, morphology, pathogenicity, phylogenetic analysis

## Abstract

Fusarium wilt pathogens represent an ongoing threat to pepper production worldwide. This is the first report providing data on the molecular identification of *Fusarium* fungi that cause wilt in pepper in the southern regions of Russia. Monitoring of the *Fusarium* infection on pepper was carried out in 2019–2022 in two economically important regions of this culture production: the Krasnodar Krai and Crimea. Based on a phylogenetic analysis of the translation elongation factor (*EF1a*) and the internal transcribed spacer (ITS), as well as the macro- and micromorphological characteristics of the fungi, the causative agents of Fusarium wilt have been identified. The causative agents identified as representatives of the *Fusarium* species composition included: *F. clavus*, *F. solani*, *F. oxysporum*, *F. verticillioides*, *F. commune*, *F. torulosum*, and *F. sporotrichioides*. Depending on the region, the specifics of biodiversity and the ratio of these species in pathocomplexes were noted. In Crimea, wilting could be attributed to all of the identified species; in the Krasnodar Krai, *F. verticillioides* and *F. clavus* were found to contribute to wilting. The pathogenicity test showed that the pathogens of pepper wilting in Russia, in addition to the already known *F. oxysporum* and *F. solani*, are the species *F. clavus* and *F. verticillioides*. This is the first report on the ability of these species to cause Fusarium wilt in pepper cultures. The obtained data will be of practical value for the development of biological control measures for fungi of the genus *Fusarium*, which cause pepper wilt in areas of industrial production and seed production. In addition, data on species composition and aggressive isolates will be used in a pepper breeding program for resistance to Fusarium wilt.

## 1. Introduction

Pepper (*Capsicum annuum* L.) is a widely grown and important vegetable crop in Russia due to its high nutritional value, unique vitamin content, and diverse mineral composition [1,2,3]. The largest producers of pepper are China, Mexico, Turkey, and Indonesia [4]. In Russia in 2020, sweet pepper occupied 44.1 hectares, with an average yield of 12.4 t/ha and the gross yield was 549.0 t [5]. In Russia, the seed-growing and production of pepper is carried out in the North Caucasus, Southern federal districts, and the Volga region, which meet the biological requirements of this crop. In epiphytological terms, these areas belong to the zone of natural focus of various phytopathogens, so the production of pepper is often unprofitable [6].

To date, this culture is exposed to various pathogens of fungal etiology; the most harmful and reported worldwide are *Fusarium* spp., *Verticillium dahliae*, *Phytophthora capsici*, *Macrophomina phaseolina*, *Rhizoctonia solani*, and *Pythium aphanidermatum* [7,8,9,10]. Among them, *Fusarium* spp. That cause wilt, spotting, and fruit rot are an economic problem threatening pepper production in many countries and Russia [11]. According to various estimates, pepper crop losses from the Fusarium wilt range from 10% to 80%, depending on the weather conditions of the year, the climatic zone, and the set of cultivated varieties [12,13]. Many *Fusarium* species can synthesize and accumulate various secondary toxic metabolites–mycotoxins in plant tissues and processed products, so they pose a serious risk to product safety [14].

*Fusarium* species exhibit great genetic and pathogenic variability. The structure of this genus is based on monophyletic clades called species complex (SC) [15]. At this time, the genus *Fusarium* includes more than 400 phylogenetic species, distributed among 23 SC [15,16]. In studies on the correct identification of fungi of the genus *Fusarium*, the most phylogenetically informative markers are the nucleotide sequences of the *EF1α* gene coding the translation elongation factor 1 alpha [17,18], the *RPB2* gene coding the RNA polymerase II second largest subunit [19], and the *CYP51C* gene coding the sterol 14-demethylase [20]. Nucleotide sequences of internal transcribed spacers (ITS) of ribosomal RNA genes are currently less used for *Fusarium* species identification. Nevertheless, in view of easy amplification and the extensive data available for comparison in public databases, this marker is useful for distinguishing numerous *Fusarium* SC, as well as for obtaining reliable genus-level identification for *Fusarium*; thus, ITS-based markers may be successfully used as additional DNA markers in the phylogenetic analysis of *Fusarium* species [21,22].

In many countries around the world, Fusarium wilt is most often caused by the most economically important species *F. oxysporum* var. *capsici* and *F. solani* [23,24,25,26,27,28,29,30]. In recent years, some researchers have proposed separating the species *F. solani* from the taxon *Fusarium* into the taxon *Neocosmospora sensu* [31]. Other researchers object to this and provide arguments confirming the stable monophyly of the genus *Fusarium* [16].

An interesting fact is that the harmfulness of species that were previously considered weakly pathogenic for crops of the Solonaceae family is increasing. For example, the species *F. equiseti* in Mexico and the Kashmir Valley caused symptoms of vascular wilting of pepper and fruit rot, leading to yield losses of up to 50% [9]. The authors point out that such losses are associated with the underestimation of this species as pathogenic. According to numerous reports of researchers, along with the dominant species *F. oxysporum* var. *carcisi* and *F. solani, F. verticillioides, F. pallidoroseum* [13,29], *F. equiseti* [9,32], *F. proliferatum,* and *F. anthophilum* [33] also participate in the development of Fusarium wilt.

To effectively combat Fusarium wilt on pepper, in addition to identifying *Fusarium* species, it is of particular importance to study the level of their pathogenicity in mycocenoses associated with this disease in order to monitor spatial and temporal changes of this characteristic [34,35]. At present, in Russia, there are no data on the current composition of the causative agents of Fusarium wilt on pepper. The goal of the current study was the species identification of fungi of the genus *Fusarium* that cause wilt of pepper in the southern regions of Russia, based on morphological and molecular characteristics, as well as the assessment of their pathogenicity for pepper in accordance with Koch’s postulates.

## 2. Materials and Methods

### 2.1. Collection of Plant Material and Isolation of Fungi

In 2019–2022, pepper plants in the phase of technical and biological ripeness of fruits with symptoms of vascular wilting were collected in two southern regions of Russia: the Krasnodar Krai (Temryuk region) and the Republic of Crimea (Simferopol region). The climate of both research areas is similar to the temperate continental climate, with unstable and insufficient moisture. The average annual precipitation in the Krasnodar Krai (Temryuk region) is 460 mm, and in the foothills of the Crimea—about 350 mm. Winters are relatively warm and short, and summers are long and hot with periods of severe drought. The long-term average annual air temperature is +9…+12 °C. The summer temperatures (July–August) range from +22 °C to +24 °C.

The percentage of spread of the disease was calculated by the formula:

$$Percent\;disease\;incidence=\frac{Total\;number\;of\;diseased\;plants}{Total\;number\;of\;examined\;plants}*100$$

To isolate the fungi, the affected tissues at the border of the affected and healthy areas were cut into several small segments, subjected to surface sterilization in 70% ethanol, washed in sterile water, dried, and then placed on potato dextrose agar (PDA) and Czapek-Dox medium prior to cultivation at a temperature of 25 °C for 3–5 days. Each grown colony was transferred by the tip of the hyphae to a new medium to obtain a pure culture of the isolate, allowing monospore colonies to be grown.

A total of 74 *Fusarium* isolates causing systemic damage on pepper were tested for pathogenicity to confirm Koch’s postulates. Subsequently, 14 isolates were selected as representatives of the species, geographical regions, and various degrees of aggressiveness. These isolates were included in the morphological characterization and phylogenetic analysis (Table 1).

### 2.2. Macro- and Micromorphology of Fusarium Isolates

The macro- and micromorphological characteristics of monospore cultures were evaluated when growing mushrooms on nutrient-agarized media of various compositions: Czapek-Dox, potato dextrose agar (PDA), and Spezieller Nährstoffarmer agar (SNA). The isolates were cultured for 7 days at a constant temperature of 25 °C and a photoperiod of 16 h/8 h (day/night). The morphology of colonies, the appearance of pigmentation, and the growth rate of fungi were evaluated daily on the studied media, recording the time of the beginning of growth (the appearance of mycelium outside the seed disk). Pigmentation changes were observed for up to 21 days. The diameter of each colony was measured in two perpendicular directions in three repetitions. The microscopic characteristics of monospore cultures were studied and recorded using a Zeiss Axio Lab A1 microscope (ZEISS, Jena, Germany) and ADF Image Capture software (version x64, 4.11.21522.20221011). At least 30–40 microstructures (conidia, chlamydospores) were measured for each isolate. The taxonomic status of *Fusarium* fungi was determined according to [36,37], including those from scientific publications as well.

### 2.3. DNA Extraction and PCR

The air mycelium of pathogens was collected from Petri dishes and moved to 1.5 mL Eppendorf tubes. Further DNA isolation was carried out using the CTAB method with various modifications [38]. In order to remove the remnants of the medium, which, to one degree or another, were captured during the selection of mycelium, the isolated samples were additionally purified with a ColGen kit (Syntol, Moscow, Russia). The DNA purity was assessed using a NanoDrop device (Thermo Fisher Scientific, Waltham, MA, USA) and the concentration was measured using a Qubit 4 device (Thermo Fisher Scientific, USA).

Based on the literature data, we selected the following primers, presented below in Table 2. The amplification program for all pairs of primers was the same: denaturation at 95 °C for 180 s (35 cycles), denaturation at 95 °C for 20 s, annealing at t* °C (Table 2) for 20 s, elongation at 72 °C for 20 s, and final elongation at 72 °C for 300 s. The PCR mixture with a volume of 50 µL contained 1 µL of a DNA solution with a concentration of 3–5 ng, 20 pM of each primer, 0.8 µL of Taq polymerase, 21.2 µL of ddH_2_O, 25 µL of a finished reaction mixture (2X) (Synthol, Russia) containing dNTP, buffer, and 3 mM MgCl_2_.

Nucleic acids were separated in agarose gel using electrophoresis [39]. The horizontal cameras of the Sub-Gell GT System and the PowerPac HC power supply (BioRad, Hercules, CA, USA) were used for the analysis.

The visualization of the results was performed on a GelDoc XR+ System transilluminator (Bio Rad, USA). The excision of PCR products from the gel was performed on an ECX-M transilluminator (VilberLourmat, Eberhardzell, Germany). The samples were also purified with a Golden kit and sequenced at Syntol. Sequence alignment was carried out in the ClustalW program [40]. 

### 2.4. Phylogenetic Analysis

Sequence alignment was carried out in the ClustalW program [41]. The sequences of type and representing strains from the NCBI GenBank database were included in the phylogenetic analysis. The determination of the best-fitting substitution model and the maximum likelihood analysis for data sets for each locus were conducted with the MEGA X 10.1 program [42]. The 561, 565, and 757 models were chosen for the analysis of ITS, SSU, and *EF1a* loci, respectively. Nodal support was assessed by a bootstrap analysis based on 1000 replicates. Sequence data were deposited in GenBank. The reference strains used for *EF1* phylogeny are presented in Supplementary S1.

### 2.5. Pathogenicity Test

A total of 74 *Fusarium* isolates causing systemic damage were tested for pathogenicity in order to confirm Koch’s postulates. At the first stage, the study of the pathogenic properties of *Fusarium* fungi was carried out using the infection of separated organs—cut young shoots of the second and subsequent orders. In the experiment, thirty varieties of pepper were used in a three-fold repetition (n = 3), with four shoots of each variety in one repetition. A spore suspension was prepared from fungi grown on PDA medium (concentration: 10^6^ spores/mL). The cut shoots, superficially sterilized with 70% ethanol, were washed in sterile water, dried, and placed in sterile buckets or test tubes with a spore suspension. Control shoots were placed in sterile water. Symptoms (wilting and falling of leaves) were recorded in dynamics and a point assessment was performed on the third, fifth, and seventh days post-inoculation (dpi). Based on the assessment, the isolates were differentiated by the degree of aggressiveness and virulence.

At the next stage, pepper seedlings were infected with moderately and highly aggressive isolates, affecting more than 30% of the tested varieties at the first stage. Ten varieties of pepper were used in the experiment in a three-fold repetition (n = 3), with ten plants of each variety in one repetition. The experiment included one variety (Podarok Moldova, Moldova), one hybrid (Knyazhichi F_1_, Russia), and eight FSBSI FSVC breeding lines (Pr-Vn-1-13-56, Pr-Vn-12-15-17, Pr-Vn-4-13-9, Pr-Vn-1-20-53, Pr-Vn-1-44-52, Pr-Vn-1-74-18, Pr-Vn-1-29-34, Pr-Vn-5-20-19). The Podarok Moldova variety (susceptible) and the F1 Knyazhich hybrid (resistant) showed a different degree of resistance under conditions of natural infection. In seedlings grown for 14 days, the roots were washed, pruned by 2 cm, and placed in mycelialspore suspensions of *Fusarium* isolates for 30 min at a concentration of 10^6^ spores per ml. Infected seedlings were transferred into cassettes with a sterile peat–perlite mixture in a ratio of 2:1 (volume of 1 cell: 160 mL). All inoculated plants were grown in a vegetative box with a relative humidity of 85%, temperature of 27 °C, and alternating lighting mode of 18 h/6 h (day/night). The choice of such environmental parameters is justified by the maximum approximation of the conditions of natural pathogenesis in the southern regions. Observations of the development of seedlings and the symptoms of the lesion were carried out in dynamics at 7, 14, and 21 dpi, calculating the disease severity index (DSI) according to the following formula according to [42]:

$$Disease\;severity\;index\left(DSI\right)=\frac{\sum \left(Score\;Amount\;of\;plants\times Corresponding\;score\right)}{Maximum\;score\;*Total\;number\;of\;plants}*100$$

**Figure 1:**
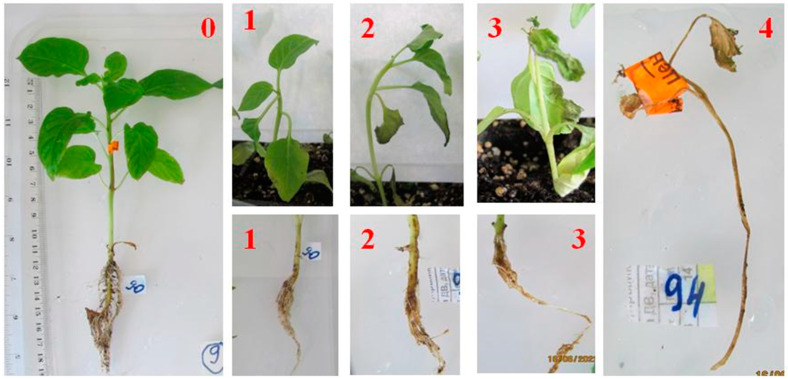
Scale used to assess the symptoms of damage to the vegetative organs (**upper row**) and root system (**lower row**) of pepper seedlings when infected with *Fusarium* isolates (the figure corresponds to a certain score).

This assessment was carried out using a four-point scale [43], which was modified for the purposes of this study (Figure 1):

- 0: The absence of symptoms.
- 1: The taproot is slightly brown and the adventitious roots and vegetative part show no symptoms of the lesion.
- 2: The taproot is brown, the adventitious roots have visible damage, the growth of the vegetative part is limited, and there is a slight wilting.
- 3: The taproot is completely brown, the appendages are poorly developed, the growth of the stem is 50% behind the control, and there is significant wilting.
- 4: The taproot is dark brown, completely necrotic or may not have formed at all, there are no adventitious roots, and the plants are completely necrotic or withered.

When gaining access to the aggressiveness of strains for vegetating plants, biometric parameters such as root length and stem height were measured in seedlings in the experimental and control variants. The aggressiveness of the studied strains was determined by the effect of exposure (EA) on the root and stem, which was calculated using the following formula.

The biometric parameters of seedlings were also measured in experimental and control variants (root length and stem height). The specifics of the effect of the studied micromycetes isolates on the biometric parameters of seedlings were judged by the magnitude of the effect of action (EA) on the root and stem, which was calculated using the following formula according to [43]:

$$Effect\;of\;action(EA)=\frac{Indicator\;in\;the\;experimental\;version-Indicator\;in\;the\;control\;version}{Indicator\;in\;the\;control\;version}*100$$

A negative value of EA indicates the inhibition of growth after infection; a positive value indicates the stimulation of the studied indicator relative to control.

Based on the degree of development of the disease and the impact on the growth and development of seedlings, mushroom isolates were differentiated into the following:

- Weakly aggressive (WA): no more than 10% of the studied varieties could be characterized as being in stages 3–4 of the four-point scale.
- Moderately aggressive (MA): 25–50% of the studied varieties could be characterized as being in stages 3–4 of the four-point scale.
- Highly aggressive (HA): 75–100% of the studied varieties could be characterized as being in stages 3–4 of the four-point scale.

### 2.6. Statistical Processing

Experimental data analysis and statistical evaluation were performed in Microsoft Excel 2016 for Windows 10 and Statistical 7.0. In order to determine the significance of difference in the aggressiveness of *Fusarium* strains, Duncan’s test was used with a probability of 95% (*p* ≤ 0.05). The influence of factors on the damage of different pepper varieties was assessed using ANOVA.

## 3. Results

### 3.1. Phytopathological Monitoring and Symptoms

**Figure 2:**
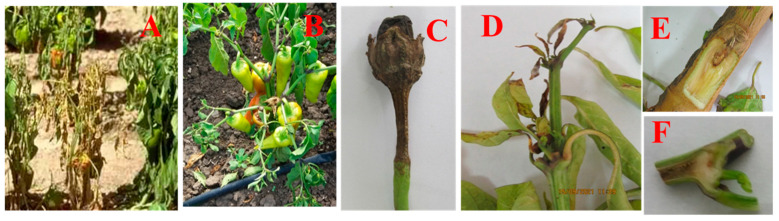
Manifestation of symptoms of withering and necrotization of the vascular system of pepper culture in the conditions of the southern regions of Russia: (**A**,**C**,**E**)—Simferopol region; (**B**,**D**,**F**)—Krasnodar Krai.

As a result of phytopathological monitoring conducted in the Republic of Crimea (Simferopol region) (2019–2022) and the Krasnodar Krai (Temryuk region) (2021–2022), it was revealed that the nature of the symptoms demonstrated by the affected pepper plants differed slightly depending on the analyzed region. In both areas, the first signs of fusarium were manifested in the budding phase with visible wilting; yellowing began with the lower leaves and gradually spread to the upper parts, and in the lower part of the root zone, stem darkening was observed on susceptible varieties (Figure 2). When the stem was cut, darkening or severe necrotization was noted.

In the conditions of Simferopol, starting from 2021, there was significant necrotization not only of the conducting system of the plants, but also of the generative organs (Figure 2C). There were often no fruits on such plants or atrophied fruits were formed. The degree of spread of tracheomycosis wilting in Simferopol was 25–64% with a lesion index of 0–4 points, depending on the conditions of the year and the level of resistance of the varieties. In the Krasnodar Krai, these indicators were significantly lower: 15–22% with a lesion index of 0–2 points.

The pathogenic complex of micromycetes isolated from plants with signs of wilting was mainly represented by fungi of the genus *Fusarium* spp.; there were also species from the genera *Alternaria* spp., *Trichoderma* spp., and *Rhizoctonia* sp., and with very severe damage-concomitant species of the genera *Aspergillus* spp., *Penicillium* spp., *Cladosporium* spp., and *Rhyzopus* spp. In total, 88 isolates of micromycetes were isolated from the affected stems and root system, with fungi of the genus *Fusarium* spp. dominating; 72 isolates were identified, which accounted for 72% of the total number of fungi studied.

All isolated *Fusarium* isolates, depending on the region of isolation and primary macro- and micromorphological characteristics, were grouped into several groups of similar morphotypes with the same characteristics. In each of the groups, the most typical isolates were identified, which were included in this study for molecular and phylogenetic identification (14 isolates). All 74 isolates of these genera were included in the experiment to study the level of aggressiveness.

### 3.2. Phylogenetic Analysis of Fungi Fusarium

**Figure 3:**
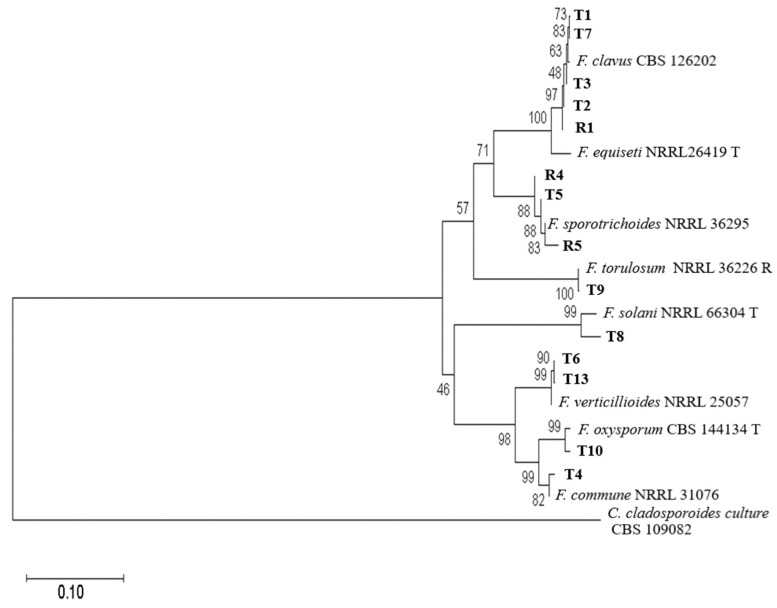
Maximum likelihood phylogenetic tree based on DNA sequence data *EF1a* locus of *Fusarium* strains. The bootstrap support values > 70% are shown at the nodes. The tree was rooted on sequences of *Cladosporium cladosporioides* strain CBS 109082. T, ex-type strain.

The species of the studied samples were determined by comparing the obtained nucleotide sequences with the annotated sequences of loci *EF1*, ITS, and SSU in various species of the genus *Fusarium* from the NCBI database. The results of the analysis are shown in Figure 3 in the form of a dendrogram. As can be seen from this dendrogram, it is possible to unambiguously determine the species of the studied strains, since the sequences of all three analyzed loci were clustered with the reference sequences of these loci of the same species. The strains F-5-21(R4), F-19-21(R5), and F-17-21(T5) belong to the species *F. sporotrichioides*; strains F-53-22(T6) and F-14-22(T13) belong to the species *F. verticillioides*; strains F-14-20 (T1), F-29-20(T2), F-38-21(T3), F-56-21(T7), and F-23-20(R1) belong to the species *F. clavus*; strain F-41-19(T4) belongs to the species *F. commune*; strain F-28-19(T10) belongs to the species *F. oxysporum*; and strains F-14-20(T8) and F-15-22(T9) belong to the species *F. solani* and *F. torulosum*, respectively.

The final determination of the species of the studied strains was carried out on the basis of a set of data obtained as a result of molecular phylogenetic and macro-micromorphological analysis.

Thus, seven species, *F. clavus*, *F. oxysporum*, *F. commune*, *F. solani*, *F. verticillioides*, *F. torulosum*, and *F. sporotrichioides*, were identified in the structure of the pathocomplex of Fusarium wilt pathogens in the Krasnodar Krai and Simferopol region.

### 3.3. Geographical Distribution of Different Fusarium Species in the Southern Regions

In the composition of the isolated and analyzed entire pathocomplex of fungi causing tracheomycosis wilting in pepper, the dominant species were *F. clavus* (46% of the total), *F. verticillioides* (20%), and *F. sporotrichioides* (16%). Depending on the region of cultivation of the crop and the affected organ of the plant being analyzed, clearly-expressed specificity in the biodiversity of the identified species and their different ratios in the studied pathocomplexes were revealed, which ultimately influenced the nature of the symptoms and the prevalence of the disease.

**Figure 4:**
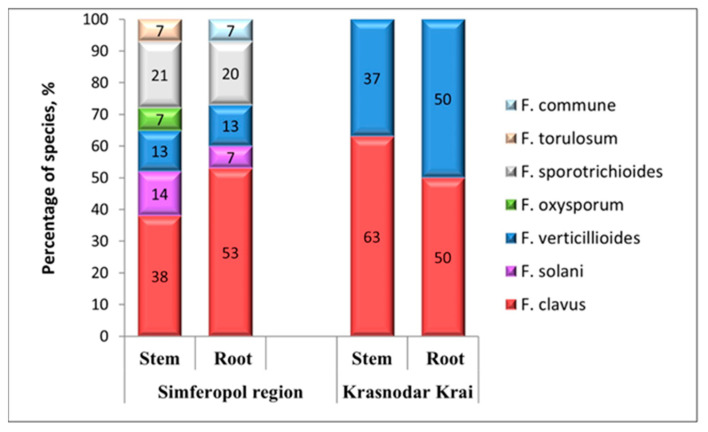
Percentages of the structure of the pathocomplex of *Fusarium* fungi isolated from pepper in the southern regions of the Russian Federation, depending on the localization of infection (2019–2022).

All identified species participated in the pathogenesis of tracheomycosis wilting of pepper plants in the Simferopol region; only their percentage ratio differed (Figure 4). The largest proportions of *Fusarium* fungi isolated from the affected pepper stems were *F. clavus, F. sporotrichioides, F. solani,* and *F. verticillioides*, constituting 38%, 21%, 14%, and 13%, respectively. The dominant species in the pathocomplex of affected roots in Simferopol were *F. clavus* (53%), *F. sporotrichioides* (20%), and *F. verticillioides* (13%). The species *F. oxysporum*, *F. torulosum*, and *F. commune* were isolated in an equal ratio (7%). *F. oxysporum* and *F. torulosum* were isolated from the affected pepper stems, and *F. commune* was isolated from the roots.

In the Krasnodar Krai, the complex of species isolated from the analyzed affected organs is less diverse and is represented by two species: *F. clavus* and *F. verticillioides*. Their ratio among the isolates from the roots in this region was equal: 50% each on the stem, with *F. clavus* dominating (63%).

Annual phytomonitoring of the species composition of Fusarium wilt pathogens on pepper allowed us to not only determine its structure, but also to identify the change in dominant species in the Simferopol region over time. In 2019, the pathocomplex of *Fusarium* fungi on pepper was represented by the species *F. solani*, *F. sporotrichioides*, *F. verticillioides*, and *F. clavus*, with a significant prevalence of *F. sporotrichioides*. By 2022, there was a significant change in the species composition in the mycocenosis: together with the previously identified species, *F. commune*, *F. oxysporum*, and *F. torulosum* began to occur. As a result, by 2022, the frequency of occurrence of *F. solani*, *F. sporotrichioides*, and *F. verticillioides* abruptly decreased, and the number of isolated isolates, depending on the species, decreased by 1.5–6 times. The niche of the dominant species causing tracheomycosis wilting in pepper culture in the Simferopol region has recently been occupied by *F. clavus, F. oxysporum,* and *F. torulosum*, with a significant dominance of *F. clavus.*

### 3.4. Study of Pathogenic Properties of Fusarium Fungi

**Figure 5:**
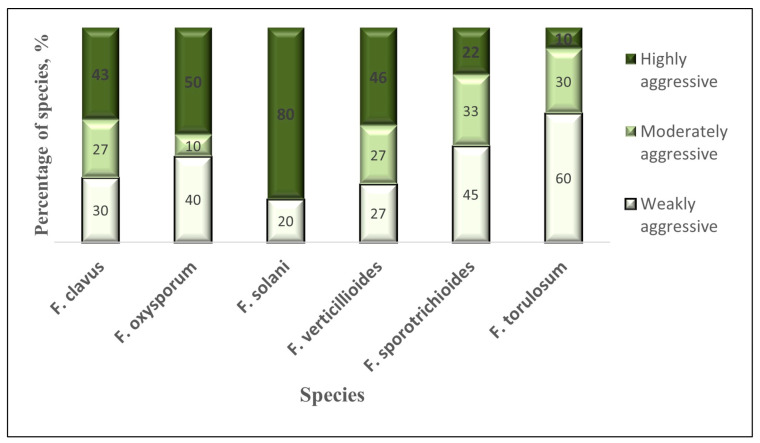
Distribution of the structure of *Fusarium* fungi with different pathogenicity levels when infecting young pepper shoots.

A total of 74 isolates of the following identified species were included in the studies of the primary pathogenicity assessment of *Fusarium* fungi: *F. clavus* (30 isolates), *F. oxysporum* (10 isolates), *F. solani* (5 isolates), *F. verticillioides* (11 isolates), *F. torulosum* (8 isolates), *F. commune* (1 isolat), and *F. sporotrichioides* (9 isolates). As a result, classes of isolates differing in pathogenicity were identified within each *Fusarium* species population. The percentage of extreme classes differed depending on the type of *Fusarium* (Figure 5).

The highest proportion of highly aggressive isolates in relation to pepper shoots was in the fungi *F. solani*, exceeding the number of weakly aggressive isolates in these species by four times. The largest number of weakly aggressive isolates against the host plant was observed in the species *F. torulosum* and *F. sporotrichioides* (45–60%, depending on the species). In the population of *F. oxysporum*, the number of species contrasting in pathogenicity was distributed in an equal ratio (40–50%). The *F. commune* isolate was weakly aggressive on pepper sprouts.

In order to focus on the differences between pathogenicity-differing isolates of fungi within species populations, data on the studied varieties and replications were averaged (Table 3). All highly aggressive isolates within the analyzed *Fusarium* species were able to cause wilting of the separated apical parts of pepper shoots to varying degrees. Shoots placed in a spore suspension showed symptoms of wilting and leaf fall, whereas control plants did not have them. The isolates of *F. solani* and *F. clavus* isolated in a separate group (*p* ≤ 0.05) had the greatest aggressiveness according to the results of this assessment. The inoculation of separated parts of pepper plants with weakly pathogenic isolates of different types (F-41-19(T4), F-15-22(T9), F-38-21, F-17-21, F-29-20, F-31-22, and F-21-20) did not cause characteristic symptoms of lesions in most of the studied samples. Moreover, although some varieties showed symptoms of wilting, in general, this group of isolates did not significantly differ from the control group (*p* ≤ 0.05).

**Figure 6:**
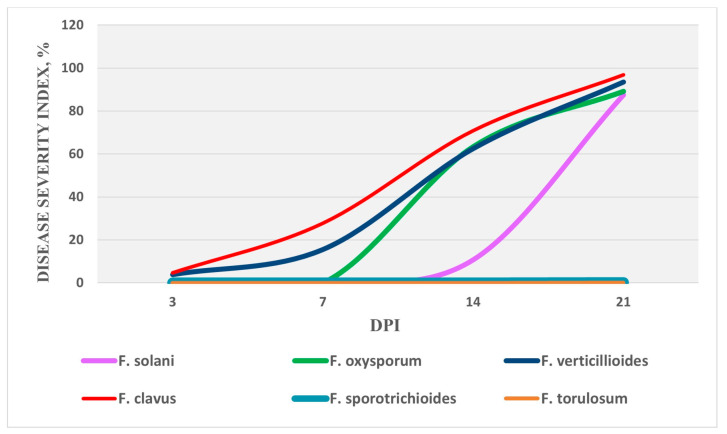
The dynamics of the onset of wilting symptoms when infected with various isolates of vegetative pepper plants. Ten varieties of pepper were included in the study; the lesion index on the graph is calculated based on the average of all analyzed varieties and three repeats.

The most aggressive isolates from each identified species were included in the second stage of studies to study the level of pathogenicity by infecting vegetative plants to identify the true pathogens of wilting that can cause infection of the root and vascular system of pepper plants in vivo. A total of seven isolates were included in the study at this stage. The study of the dynamics of the disease revealed differences between highly aggressive isolates in the timing of the appearance of the first symptoms (Figure 6). When infected with *F. clavus* and *F. verticillioides*, the first signs of wilting and leaf fall in seedlings of highly susceptible samples were noted as early as day 3, and the overall increase in the degree of disease development across the entire population of the studied varieties during the analyzed period was almost linear. In seedlings inoculated with *F. solani* and *F. oxysporum* species, the symptoms of wilting and growth retardation relative to the control ones appeared later, on days 7 and 14, respectively, and were characterized by more rapid exponential development of the disease after the first symptoms appeared.

By the time of the final accounting on day 21, a significantly (*p* ≤ 0.05) high aggressiveness of *F. solani, F. oxysporum, F. clavus*, and *F. verticillioides* was detected in relation to the tested pepper varieties (Table 4). During the experiment, a high degree of disease development was noted with the inoculation of these species (87.5–93.5%) with respect to the overwhelming number of tested varieties and the inhibition of the development of the root system (EA= −24…−41%), as well as the development of the above-ground parts of the seedlings (EA= −22…−44%).

**Figure 7:**
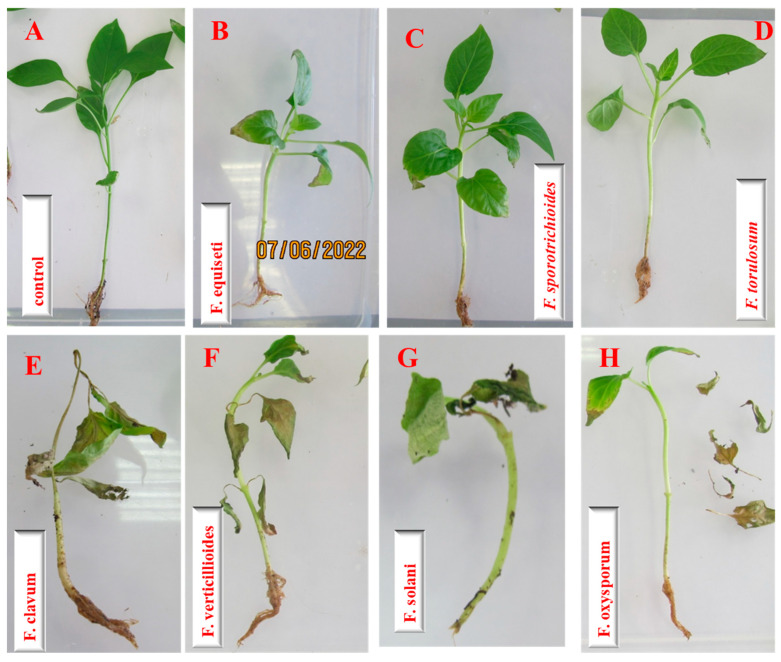
Symptoms of infection of feather seedlings, foreign cultural modifications: (**A**)—control (sterile water); (**B**)—*F. sporotrichioides*; (**C**)—*F. torulosum*; (**D**)—*F. verticillioides*; (**E**)—*F. solani;* (**F**)—*F. oxysporum;* (**G**)—*F. clavus*.

When pepper plants were inoculated with *F. sporotrichioides* and *F. torulosum*, no clear external symptoms were observed during the experiment. Although plants with signs of wilting were found for some susceptible varieties, on average, for all of the analyzed plants, the differences relative to the control variants were insignificant. The most aggressive species were *F. clavus* and *F. verticillioides*, which have a similar type and nature of symptoms in infected plants, as well as a more significant degree of disease development (*p* ≤ 0.05). The symptoms of wilting in pepper seedlings after inoculation with various *Fusarium* species are shown in Figure 7.

Due to the fact that a wide range of pepper varieties with varying degrees of resistance to the studied species were included in this study (disease development: 12.5–100%, depending on the variety), ANOVA analysis showed the significant impact of both the *Fusarium* species and pepper variety on the outcome of infection, with differences only in the magnitude of the effect. The strongest effect (69.2% dispersion) was observed for the mushroom species, with 23.6% dispersion for the variety and 7.7% dispersion for their interaction.

### 3.5. Morphological Description of Fusarium Species Associated with Pepper wilt in the Southern Regions of the Russian Federation

**Figure 8:**
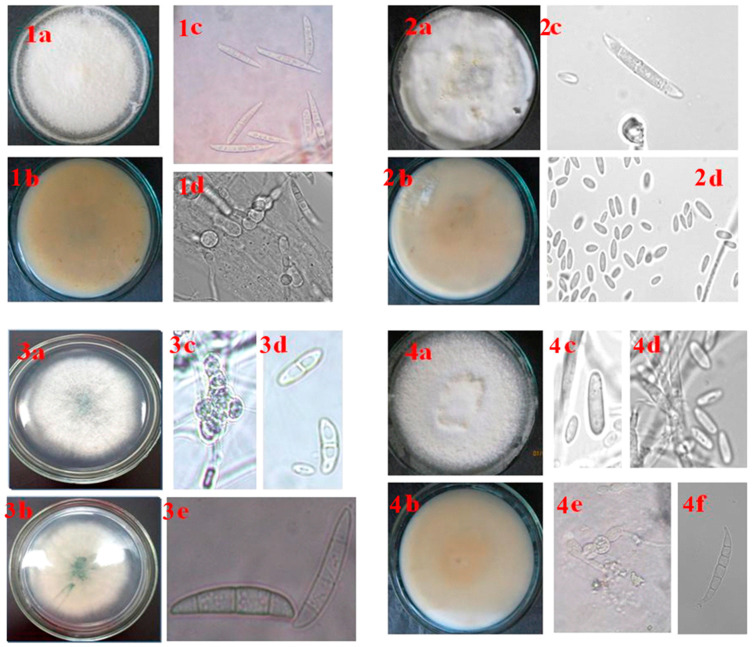
Colonies and micromorphological features of *Fusarium* species causing Fusarium wilt of pepper (PDA at 25°C, 16h light/8h dark, 7 days); scale bars represent 20 μm; *F. clavus*: (**1a**,**1b**)—obverse and reverse; (**1c**)—macroconidia; (**1d**)—chlamydospores; *F. verticillioides*: (**2a**,**2b**)—obverse and reverse; (**2c**)—macroconidia, (**2d**)—microconidia; *F. solani*: (**3a**,**3b**)—obverse and reverse; (**3c**)—chlamydospores; (**3d**)—microconidia; (**3e**)—macroconidia; *F. oxysporum*: (**4a**,**4b**)—obverse and reverse; (**4c**)—mesoconidia; (**4d**)—microconidia; (**4e**)—chlamydospores; (**4f**)—macroconidia.

The appearance of colonies and micromorphological characteristics of *Fusarium* fungi species causing wilting in pepper plants in the southern regions of Russia are shown in Figure 8, Table 5.

## 4. Discussion

The main objective of this work was to study the genetic diversity of *Fusarium* fungi on pepper culture in the southern regions of Russia. Another important aspect of this study was to identify the true pathogens associated with tracheomycosis wilting of pepper in the south of Russia. We used the internal transcribed spacer as the primary definition, as they are suitable for determining the genus and species complex of pathogens. The *EF1a* gene, due to its greater conservatism and accuracy, was used to determine the species. The use of a combination of molecular genetic and morphological approaches made it possible, for the first time in Russia, to determine the taxonomic affiliation of the most common and harmful pathogens of Fusarium wilt in pepper cultures. Seven species, *F. clavus*, *F. oxysporum*, *F. commune, F. solani*, *F. verticillioides*, *F. torulosum*, and *F. sporotrichioides*, have been identified and described within the complex of pathogens causing Fusarium wilt of pepper in the Krasnodar Krai and Simferopol regions. Among the 74 *Fusarium* isolates analyzed, the dominant species were *F. clavus* (46%), *F. verticillioides* (20%), and *F. sporotrichioides* (16%). Difficulties with clearly identifying the species of some isolates are probably objective, since the species *F. equiseti*, *F. incarnatum*, and *F. clavus* belong to the same complex of *F. incarnatum-equiseti* species [44,45] and it is not surprising that they turned out to be largely similar to each other, at least at the level of sequences of the studied loci. The species *F. sporotrichioides, F. verticillioides, F. solani, F. torulosum*, and *F. oxysporum* belong to different species complexes that differ quite significantly from each other at the molecular level; therefore, they can be easily distinguished using molecular genetic analysis [16,46]. The final determination of the species of the studied samples should be carried out on the basis of a set of data obtained as a result of molecular phylogenetic and macro–micromorphological analysis.

It is known that the nature and extent of the manifestation of external signs of the disease, especially with complex pathogen damage, are determined by examining the distribution of species composition in the pathocomplex, location of infection, degree of aggressiveness and features of the relationship of pathogens in a particular mycosociety, level of plant resistance, and influence of external environmental factors [35,47]. In this study, it was shown that all identified species can contribute to the pathogenesis of tracheomycosis wilting of pepper plants in the Republic of Crimea. An interesting fact is that in this region, during the four years under study, there was not only an expansion of species diversity, but also a change in the *Fusarium* species prevailing in the pathocomplex. In the Krasnodar Krai, the species complex is less diverse and is represented by two species: *F. clavus* and *F. verticillioides*. We believe that the list of *Fusarium* species associated with pepper wilting in this region is still incomplete, which requires further research.

The ability of *F. oxysporum* (FOSC) and *F. solani* (FSSC), as the main pathogens, to cause symptoms of vascular wilt in plants of the Solonaceae family has also been noted by other scientists in their studies. Recent studies conducted in North Carolina [47], Mexico [48], Poland [49], South Korea [50], Pakistan [29], and India [15] have shown that these two dominant pathogens have remained a serious threat to pepper, tomato, physalis, eggplant, and potato production in these regions.

In recent years, there has been evidence of an increase in the harmfulness and contribution of *F. clavus* species belonging to the *F. incarnatum-equiseti* species complex (SC) (FIESC) in the pathogenesis of not only the rot of fruits and leaf spots on agricultural plants, but also vascular wilt. In Thailand [51], the species *F. clavus*, together with a representative of *F. tricinctum* SC, caused dirty panicle disease in coconuts. In addition, this species has recently been identified in Italy on tomato plants with symptoms of wilting and fruit rot [52], in Tunisia on a date palm with symptoms of wilting [53], in Algeria on potatoes with symptoms of wilting and dry rot [53], in Tunisia on roses with symptoms of wilting [54], and on vegetable cultivars with symptoms of spotting [55]. However, other researchers report that *F. clavus* is an endophytic fungus, the colonization of which enabled the mitigation of the harmful effects of salt stress in muskmelon seedlings [56].

All the information available in the literature regarding *F. verticillioides*, which is a representative of *F. fujikuroi* SC (FFSC), is related to the study of this pathogen as one of the main pathogens of the rot of corn stalks and cereals [57]. It is noted that *F. verticillioides* has the ability to produce mycotoxins of fumonisin, leading to human and animal diseases [58]. There are limited data devoted to research on the ability of this species to cause wilting in vegetable crops. Thus, the contribution of this species in the pathogenesis of Fusarium wilt in pepper in Pakistan was reported [33].

Our study also noted the high aggressiveness and wide virulence of *F. solani*, *F. oxysporum*, *F. clavus*, and *F. verticillioides* in relation to a large set of tested pepper varieties. During the experiment, a high degree of disease development was noted on the tested varieties of pepper with inoculation with these species (87.5–93.5%), with a significant inhibition of the development of the root system, as well as the growth and development of seedlings. Moreover, the species *F. clavus* and *F. verticillioides* had the highest aggressiveness, having a similar type of manifestation of symptoms in infected plants and the nature of its manifestation, as well as a more significant degree of disease development (*p* ≤ 0.05). As the analysis of the literature shows, this is the first report on the ability of *F. clavus* to cause Fusarium wilt in pepper.

In the conducted laboratory test, the species *F. commune*, *F. sporotrichioides*, and *F. torulosum*, when infected, could not induce wilting symptoms on the analyzed pepper plants.

Within each *Fusarium* species population, when affected in vivo, different pathogenicity classes are distinguished [59]. For the primary express assessment of pathogenicity in relation to host plants and the organ specificity of their action, it is important to choose the most effective method of infection, taking into account the characteristics of the pathogenesis caused by the pathogen. As our experience shows, separated leaves and fruits of plants can be used for the primary rapid assessment of the pathogenicity of parenchymal pathogens (spots and rot). For vascular-type diseases (wilting and necrosis of the stem and roots), this method does not always reveal this activity of the pathogen, and the most informative symptom in this case is the infection of cut young shoots, which allows us to isolate the most aggressive isolates within the analyzed populations of *Fusarium* fungi. In the future, the infection of the root systems of vegetative pepper plants with isolated aggressive isolates would make it possible to identify the true pathogens of Fusarium wilt or root rot.

## 5. Conclusions

New knowledge on the diversity and pathogenicity of *Fusarium* fungi causing pepper wilt in southern regions of Russia was obtained in this study. The species composition of *Fusarium* fungi was confirmed by the results of phylogenetic analysis, and *F. clavus*, *F. oxysporum*, *F. verticillioides*, *F. solani*, *F. commune*, *F. sporotrichioides*, and *F. torulosum* were identified. Depending on the analyzed growing region, the specificity in the biodiversity of the identified species and their different ratios in pathocomplexes were noted. When studying the organ-specific activity, as well as the aggressiveness of the isolated isolates against a wide range of pepper varieties, it was found that only the species *F. clavus*, *F. verticillioides*, *F. solani*, and *F. oxysporum* can cause symptoms of tracheomycosis wilt. For the first time, the high aggressiveness of *F. clavus* and *F. verticillioides* strains to pepper plants was demonstrated. The obtained data will be of practical value for the development of control measures for fungi of the genus *Fusarium*, which cause pepper wilt in areas of industrial production and seed production. In addition, data on species composition and aggressive isolates will be used in a pepper breeding program for resistance to Fusarium wilt.

## Figures and Tables

**Table 1:** *Fusarium* strains isolated from *Capsicum annuum* L. and included in the phylogenetic analyses.

Strain	*Fusarium* Species	Origin	Substrate	Year	ID GenBank			% Similarities EF1a *
					*ITS1/4*	SSU	EF1-a	
F-14-20 (T1)	*F. clavus*	Simferopol	stem	2022	PP033904	PP033928	PP054861	99.33
F-38-21 (T3)	*F. clavus*	Simferopol	root	2022	PP033903	PP033927	PP054862	99.66
F-56-21 (T7)	*F. clavus*	Simferopol	root	2022	PP033902	PP033926	PP054863	99.61
F-53-22 (T6)	*F. verticillioides*	Krymsk	stem	2021	PP033900	PP033924	PP054856	99.54
F-14-22 (T13)	*F. verticillioides*	Simferopol	stem	2021	PP033899	PP033923	PP054857	99.69
F-14-20 (T8)	*F. solani*	Simferopol	stem	2020	PP033908	PP033932	PP054853	95.26
F-28-19 (T10)	*F. oxysporum*	Simferopol	stem	2022	PP033907	PP033930	PP054859	98.54
F-41-19 (T4)	*F. commune*	Simferopol	root	2021	PP033906	PP033929	PP054860	98.87
F-23-20 (R1)	*F. clavus*	Krymsk	stem	2021	PP033913	PP033937	PP054850	99.5
F-29-20 (T2)	*F. clavus*	Krymsk	stem	2021	PP033912	PP033936	PP054849	99.48
F-5-21 (R4)	*F. sporotrichioides*	Simferopol	root	2021	PP033909	PP033933	PP054851	98.86
F-17-21 (T5)	*F. sporotrichioides*	Simferopol	root	2021	PP033910	PP033934	PP054854	99.19
F-19-21 (R5)	*F. sporotrichioides*	Simferopol	stem	2021	PP033905	PP033931	PP054858	98.22
F-14-22 (T9)	*F. torulosum*	Simferopol	stem	2022	PP033911	PP033935	PP054855	99.68

Note: * the similarity of obtained sequences to the sequences of the type/representative *Fusarium* strains from GenBank. https://www.ncbi.nlm.nih.gov.

**Table 2:** Sequences of primers used for amplification of ITS and *EF1a* loci.

Primer	Sequence 5′-3′	Product Size (bp)	Tm (°C)	A Source
ITS1 ITS4	F:TCCGTAGGTGAACCTGCGG R:TCCTCCGCTTATTGATATGC	~550	62*	[21]
ITS5 ITS4	F: GGAAGTAAAAGTCGTAACAAGG R:TCCTCCGCTTATTGATATGC	~550	62*	[21]
EF-1 EF-2	F:ATGGGTAAGGARGACAAGAC R:GGARGTACCAGTSATCATG	~680	55*	[19]

**Table 3:** Pathogenicity of *Fusarium* isolates differing in degree of aggressiveness against young pepper shoots (day 7).

Strains	Type	Degree of Aggressiveness *	Withering, Score **	
			Range	Average
Control			0	0 ^a^
F-41-19(T4)	*F. commune*	WA	0–0.5	0.3 ^a^
F-14-22	*F. verticillioides*	WA	0–0.5	0.5 ^a^
F-38-21	*F. clavus*	WA	0–0.5	0.5 ^a^
F-17-21	*F. sporotrichioides*	WA	0–0.5	0.2 ^a^
F-29-20	*F. clavus*	WA	0–0.5	0.2 ^a^
F-31-22	*F. torulosum*	WA	0–0.5	0.2 ^a^
F-21-20	*F. solani*	WA	0–0.5	0.2 ^a^
F-15-22(T9)	*F. torulosum*	HA	0–3.1	2.5 ^bc^
F-5-21(R4)	*F. sporotrichioides*	HA	0–3.3	2.6 ^bc^
F-28-19(T10)	*F. oxysporum*	HA	0.5–4	2.8 ^bc^
F-23-20(R1)	*F. clavus*	HA	0.5–4	3.0 ^c^
F-53-22(T6)	*F. verticillioides*	HA	0.5–4	3.2 ^cd^
F-56-21(T7)	*F. clavus*	HA	0.5–4	3.5 ^de^
F-14-20(T8)	*F. solani*	HA	0.5–4	4.0 ^e^

Note: * WA, weakly aggressive; HA, highly aggressive. ** The table shows the average values of the lesion index of all analyzed plants infected with the isolate; a–e: values with the same letter do not significantly differ with a 95% probability according to the Duncan test.

**Table 4:** The severity index of the disease and the effects of the action (EA) on the growth of seedlings of various varieties of pepper during inoculation with various types of *Fusarium*.

Strains	Type	DSI *, %		EA **, %		Proportion of *** Susceptible Samples, %
		Range	Average	Root	Stem	
Control		0	0a	0 ^a^	0 ^a^	0
F-5-21(R4)	*F. sporotrichioides*	0–5.4	0.1 ^a^	0 ^a^	0 ^a^	0
F-15-22(T9)	*F. torulosum*	0–5.4	0.1 ^a^	0 ^a^	0 ^a^	0
F-14-20(T1)	*F. solani*	12.5–100	87.5 ^b^	−28.0 ^b^	−24.0 ^b^	68.8
F-28-19(T10)	*F. oxysporum*	12.5–100	89.2 ^b^	−24.0 ^b^	−22.0 ^b^	74.5
F-56-21(T7)	*F. clavus*	12.5–100	96.8 ^bc^	−39.0 ^bc^	−40.0 ^bc^	76.0
F-53-22(T6)	*F. verticillioides*	12.5–100	93.5 ^bc^	−41.0 ^bc^	−44.0 ^bc^	76.0

Note: * DSI—disease severity index; ** EA—effect of the action on the root and stem; the table shows the average values of the degree of disease development in the aggregate of all analyzed plants infected with the isolate; a–c: values with the same letter do not reliably have a significant difference with a probability of 95% according to the Duncan test; ***—the percentage of affected samples with a degree of disease development of more than 50% (to determine the virulence of isolates).

**Table 5:** Cultural and micromorphological features of *Fusarium* strains isolated from pepper.

Characteristic	*F. verticillioides,* F-53-22(T6)	*F. oxysporum, * F-28-19(T10)	*F. solani,* F-14-20(T1)	*F. clavus,* F-56-21(T7)
growth rate on PDA, mm/day±SE	13.3 ± 0.21	14.7 ± 0.36	10.5 ± 0.30	14.3 ± 0.27
mycelium	White, dense mycelium	White, felt mycelium	White, felt mycelium	White, felt, uniform mycelium
type of conidiophores	monophialides	monophialides	monophialides	monophialides
pigment	yellowish on days 19–21	lemon on days 9–12	blue on days 5–14	lilac on days 4–5
Microconidia:				
size, µm	8.3 ± 1.1 × 2.9 ± 0.7	7.9 ± 1.1 × 2.8 ± 0.3	12.1 ± 1.1 × 4.7 ± 0.6	4.6 ± 0.6 × 2.4 ± 0.2
septation	0	0–1	0–1	0–1
shape	oval	oval	oval to slightly curved	oval to slightly curved
Macroconidia:				
size, µm	16.5 ± 2.5 × 3.1 ± 1.1	15.3 ± 4.1 × 3.2 ± 0.9	18.5 ± 0.9 × 5.9 ± 0.5	15.1 ± 0.5 × 2.8 ± 0.3
septation	3–4	3–4	2–3	3–5
shape	straight to slightly curved	falcate to almost straight	slightly curved	fusiform or slightly curved
Chlamydospores:				
size, µm	not formed	8.5 ± 0.7 × 8.5 ± 0.9	8.6 ± 0.8 × 8.6 ± 0.9	10.4 ± 0.9 × 10.1 ± 1.1
shape	not formed	globose	globose	globose
abundance	not formed	abundant, single, or in pairs	abundant, single, or in pairs	formed in large quantities of 3–4 pieces in a chain

## Data Availability

Data are contained within the article and Supplementary Materials.

## Supplementary Materials

The following are available online at https://www.mdpi.com/article/10.3390/microorganisms12020343/s1.
