# Supplementary material for: Fusarium Species Causing Pepper Wilt in Russia: Molecular Identification and Pathogenicity
Source: Microorganisms. 2024 Feb 6;12(2):343. doi: 10.3390/microorganisms12020343 (PMC10893445; doi:10.3390/microorganisms12020343)
Supplement: Supplementary file 1 [file microorganisms-12-00343-s001.zip › microorganisms-2788089-supplementary.pdf]

Supplementary S1. Information onthe type strains of *Fusarium* used in the phylogenetic study.

Fusarium species	Species complex	Strain	Source	Country	GenBankacc.tef
F. equiseti	FIESC	CBS 307.94 = NRRL 26419 T	Soil	Germany	GQ505599
F. clavus	FIESC	CBS 126202 = RMF N 38 T	Desert surface soil	Namibia	MN170456
F. commune	FNSC	CBS 110090 = NRRL 31076 T	*Pisum sativum*	Denmark	AF362263
F. oxysporum	FOSC	CBS 144134 T	Rotten potato tuber	Germany	MH485044
F. solani	FSSC	NRRL 66304 T	Potato tuber	Slovenia	KT313611
F. sporotrichioides	FSAMSC	NRRL 36295 = CBS 178.64 R	Tobacco seedling root	Belgium	MW233116
F. torulosum	FTSC	CBS 126.95 = NRRL 36226 R	*Pinus sp.*	Australia	OL772750
F. verticillioides	FFSC	CBS 125.73=ATCC 24378=NRRL 25057 R	*Trichosanthes dioica*	India	MW402012
